# Serum creatinine as a biomarker for dystrophinopathy: a cross-sectional and longitudinal study

**DOI:** 10.1186/s12883-021-02382-7

**Published:** 2021-09-25

**Authors:** Liang Wang, Min Xu, Dawei Liu, Yingyin Liang, Pinning Feng, Huan Li, Yuling Zhu, Ruojie He, Jinfu Lin, Huili Zhang, Ziyu Liao, Cheng Zhang

**Affiliations:** 1 Department of Neurology, The First Affiliated Hospital, Sun Yat-sen University, Guangdong Provincial Key Laboratory of Diagnosis and Treatment of Major Neurological Diseases, National Key Clinical Department and Key Discipline of Neurology, No. 58 Zhongshan Road 2, Guangzhou, 510080 China; 2 Department of Dermatology, The Second Affiliated Hospital of Guangzhou Medical University, No. 250 Changgang East Road, Guangzhou, 510260 China; 3 Department of Pathology, The First Affiliated Hospital, Sun Yat-sen University, No. 58 Zhongshan Road 2, Guangzhou, 510080 China; 4 Department of Laboratory, The First Affiliated Hospital, Sun Yat-sen University, No. 58 Zhongshan Road 2, Guangzhou, 510080 China; 5 Department of Neurology, Guangzhou First People’s Hospital, No. 1 Panfu Road, Guangzhou, 510180 China

**Keywords:** Dystrophinopathy, Biomarker, Disease progression, Serum creatinine

## Abstract

**Background:**

Dystrophinopathy, a common neuromuscular disorder, includes Duchenne muscular dystrophy (DMD) and Becker muscular dystrophy (BMD). Many researches are currently ongoing to develop curative approaches, which results in an urgent need for biomarkers of disease progression and treatment response. This study investigated whether the serum creatinine (SCRN) level can be used as a biomarker of disease progression in dystrophinopathy.

**Methods:**

We enrolled 377 male patients with dystrophinopathy and 520 male non-dystrophinopathy controls in a cross-sectional study. From this cohort, 113 follow-up patients were enrolled in a longitudinal study. Patients’ demographic information, motor function, muscle fatty infiltration, and muscle dystrophin levels were evaluated. We investigated correlations between these parameters and SCRN levels, and determined changes in SCRN levels with maturation and with motor function changes.

**Results:**

Our results showed SCRN levels correlated with motor function (FDR < 0.001) and timed test results (FDR between < 0.001–0.012), as well as with muscle fatty infiltration (FDR < 0.001) and dystrophin levels (FDR = 0.015 and 0.001). SCRN levels increased with maturation in control individuals; it slowly increased with maturation in patients with BMD but decreased generally with maturation in patients with DMD. The longitudinal study further demonstrated that SCRN levels were associated with motor function.

**Conclusions:**

These findings indicated that the SCRN level is a promising biomarker for assessing disease progression in dystrophinopathy and could be used as a potential outcome measure in clinical trials.

**Supplementary Information:**

The online version contains supplementary material available at 10.1186/s12883-021-02382-7.

## Background

Dystrophinopathy is a common hereditary neuromuscular disorder, characterized by limb, respiratory, and cardiac muscle weakness [1], and mainly includes Duchenne muscular dystrophy (DMD), which has a severe course, and Becker muscular dystrophy (BMD), which has a better prognosis [1]. Mutations abolishing dystrophin function lead to DMD, and those retaining partially functional dystrophin cause BMD [2]. Many novel therapeutic approaches are currently being evaluated in clinical trials [2]; consequently, effective and objective endpoints for evaluating disease progression and treatment responses are urgently needed.

The assessment of motor function is commonly used to evaluate disease progression, their applications are limited because of the dependence on moderator skill and patient compliance [3, 4]. Furthermore, muscle biopsy and magnetic resonance imaging (MRI) are valuable tools, but both have limitations: muscle biopsy is invasive, and MRI is expensive and requires sedation for young patients [5, 6]. Notably, serum creatine kinase has been used as a biomarker for diagnosis, but not for continuous therapeutic monitoring, because of its high variability [7]. Matrix metalloproteinase-9 and muscle-specific microRNAs are potential biomarkers, but their applicability remains uncertain [4, 8]. Therefore, objective biomarkers that can be used to monitor disease progression in dystrophinopathy are still needed.

Our previous study indicates that the serum creatinine (SCRN) level is a promising marker for distinguishing between DMD and BMD [9]. However, whether the SCRN level can reflect disease progression remains unknown. Accordingly, the current cross-sectional and longitudinal study tested the hypothesis that the SCRN level could aid in monitoring progression in dystrophinopathy.

## Methods

### Participants

We enrolled 377 male patients with dystrophinopathy (DMD, 283; BMD, 94), who visited our clinic between September 2012 and February 2019 at the First Affiliated Hospital, Sun Yat-sen University. Among them, 163 patients participated in our previous study [9, 10]. The diagnosis was confirmed based on either histological evaluation (*n* = 7) or genetic testing (*n* = 356), or both (*n* = 14). Of them, 113 follow-up patients (DMD, 84; BMD, 29) were included in a longitudinal study. Furthermore, 520 non-dystrophinopathy male controls with normal serum urea levels (age range: 0–25 years) visiting our hospital for other diseases (excluding neuromuscular disorder, kidney disease, diabetes mellitus, or liver disease) were also included in this study, with 20 controls for each year of age. All patients with dystrophinopathy visited the clinic because of motor dysfunction, asymptomatic/pauci-symptomatic hyperCKemia, or hyperaminotransferasemia [11]. Patients who were able to ambulate at the age of ≥16 years were classified into the BMD group, and the others into the DMD group; the latter included patients with DMD who lost ambulation before the age of 12 years and patients with intermediate muscular dystrophy (IMD) who lost ambulation at the age of 12–16 years because it is hard to distinguish between IMD and DMD in early stages [12]. Among patients who were unable to be classified in the aforementioned manner, those with the onset of motor dysfunction before the age of 5 years were categorized as having DMD, and those with no motor dysfunction before the age of 5 years and nearly normal motor function or mild motor dysfunction at the age of ≥5 years were categorized as having BMD [12–14]. Patients with normal motor functions before the age of 5 years were followed up and phenotype judgments were made when they reached 5 years of age. Detailed participant information is presented in Tables 1, 2 and 3. The follow-up information in the longitudinal study is presented in Table 4.

**Table 1:** Basic information of patients with dystrophinopathy

		Number (%)				Total number (%)	Age (y)
	0–4 y	5–9 y	10–14 y	15–19 y	20–25 y		
Ethnic	101	208	47	11	10	377	
East Asian	101 (100.00)	208 (100.00)	47 (100.00)	11 (100.00)	10 (100.00)	377 (100.00)	7 (4–9)
Clinical phenotype	101	208	47	11	10	377	
BMD	19 (18.81)	44 (21.15)	15 (31.91)	7 (63.64)	9 (90.00)	94 (24.93)	7 (5–12)
DMD	82 (81.19)	164 (78.85)	32 (68.09)	4 (36.36)	1 (10.00)	283 (75.07)	7 (4–8)
Vignos scale	78 (77.23)	205 (98.56)	46 (97.87)	11 (100.00)	9 (90.00)	349 (92.57)	7 (5–9)
Brooke scale	78 (77.23)	202 (97.12)	37 (78.72)	11 (100.00)	9 (90.00)	337 (89.39)	7 (5–9)
complete GSGC scale	18 (17.82)	45 (21.63)	19 (40.43)	5 (45.45)	3 (30.00)	90 (23.87)	7 (5–10)
Gait	42 (41.58)	107 (51.44)	33 (70.21)	7 (63.64)	4 (40.00)	193 (51.19)	7 (5–9)
Time to walk 10 m	8 (7.92)	20 (9.62)	0 (0.00)	0 (0.00)	2 (20.00)	30 (7.96)	6.5 (4–8)
Climbing stairs	24 (23.76)	54 (25.96)	27 (57.45)	6 (54.55)	3 (30.00)	114 (30.24)	7 (5–10.25)
Time to climb four standard steps	14 (13.86)	20 (9.62)	0 (0.00)	0 (0.00)	2 (20.00)	36 (9.55)	5 (4–7)
Gowers’ manoeuvre	46 (45.54)	111 (53.37)	33 (70.21)	8 (72.73)	5 (50.00)	203 (53.85)	7 (5–9)
Time to stand up from ground	41 (40.59)	66 (31.73)	7 (14.89)	2 (18.18)	3 (30.00)	119 (31.56)	6 (4–8)
Arising from a chair	18 (17.82)	51 (24.52)	24 (51.06)	5 (45.45)	3 (30.00)	101 (26.79)	8 (5–10)
Time to arise from sitting	10 (9.90)	27 (12.98)	3 (6.38)	0 (0.00)	2 (20.00)	42 (11.14)	7 (4.75–8)
Loss of ambulation	0 (0.00)	7 (3.37)	16 (34.04)	4 (36.37)	1 (10.00)	28 (7.43)	11 (9.5–13.5)
MRI in legs	5 (4.95)	21 (10.10)	4 (8.51)	0 (0.00)	0 (0.00)	30 (7.96)	6.9 ± 2.71
Muscle biopsy	3 (2.97)	12 (5.77)	3 (6.38)	1 (9.09)	2 (20.00)	21 (5.57)	8(6–10)
Glucocorticoid administration							
Oral administration	10 (9.90)	74 (35.58)	20 (42.55)	0 (0.00)	2 (20.00)	106 (28.12)	7 (6–9)
None	68 (67.33)	131 (62.98)	20 (42.55)	2 (18.18)	2 (20.00)	223 (59.15)	6 (4–8)
Unknown	23 (22.77)	3 (1.44)	7 (14.89)	9 (81.82)	6 (60.00)	48 (12.73)	5.5 (3–15)
Mutation analysis	101	204	45	11	9	370	
Deletion	68 (67.33)	138 (67.65)	27 (60.00)	6 (54.55)	6 (66.67)	245 (66.22)	6.5 (4–9)
Duplication	11 (10.89)	22 (10.78)	7 (15.56)	0 (0.00)	0 (0.00)	40 (10.81)	7.23 ± 3.18
Point mutation	22 (21.78)	44 (21.57)	11 (24.44)	5 (45.45)	3 (33.33)	85 (22.97)	7 (4–9)

*BMD* Becker muscular dystrophy, *DMD* Duchenne muscular dystrophy, *GSGC* Gait, Stairs, Gowers’ manoeuvre, Chair, *y* year. Age with normal distributions and without normal distributions were presented as mean ± standard deviation and median (interquartile range: P25–P75), respectively

**Table 2:** Basic information of patients with Duchenne muscular dystrophy

		Number (%)				Total number (%)	Age (y)
	0–4 y	5–9 y	10–14 y	15–19 y	20–25 y		
Ethnic	82	164	32	4	1	283	
East Asian	82 (100.00)	164 (100.00)	32 (100.00)	4 (100.00)	1 (100.00)	283 (100.00)	7 (4–8)
Vignos scale	62 (75.61)	162 (98.78)	31 (96.88)	4 (100.00)	1 (100.00)	260 (91.87)	7 (5–9)
Brooke scale	62 (75.61)	159 (96.95)	22 (68.75)	4 (100.00)	1 (100.00)	248 (87.63)	7 (4.5–8)
complete GSGC scale	9 (10.98)	19 (11.59)	14 (43.75)	3 (75.00)	1 (100.00)	46 (16.25)	9 (6–12)
Gait	28 (34.15)	77 (46.95)	26 (81.25)	4 (100.00)	1 (100.00)	136 (48.06)	8 (5–9)
Time to walk 10 m	7 (8.54)	12 (7.32)	0 (0.00)	0 (0.00)	0 (00.00)	19 (6.71)	6 (4–7.5)
Climbing stairs	14 (17.07)	27 (16.46)	22 (68.75)	4 (100.00)	1 (100.00)	68 (24.03)	9 (6–11)
Time to climb four standard steps	11 (13.41)	12 7.32)	0 (0.00)	0 (0.00)	0 (00.00)	23 (8.13)	5 (4–7)
Gowers’ manoeuvre	31 (37.80)	80 (48.78)	24 (75.00)	4 (100.00)	1 (100.00)	140 (49.47)	7 (5–9)
Time to stand up from ground	29 (35.37)	45 (27.44)	2 (6.25)	0 (00.00)	0 (00.00)	76 (26.86)	6 (4–7)
Arising from a chair	9 (10.98)	25 (15.24)	17 (53.13)	3 (75.00)	1 (100.00)	55 (19.43)	9 (7–11)
Time to arise from sitting	8 (9.76)	18 (10.98)	1 (3.13)	0 (0.00)	0 (00.00)	27 (9.54)	7 (4–8)
Loss of ambulation	0 (0.00)	7 (4.27)	16 (50.00)	4 (100.00)	1 (100.00)	28 (9.89)	11 (9.5–13.5)
MRI in legs	3 (3.66)	18 (10.98)	1 (3.13)	0 (0.00)	0 (00.00)	22 (7.77)	6.64 ± 2.13
Muscle biopsy	1 (1.22)	11 (6.71)	2 (6.25)	0 (0.00)	1 (100.00)	15 (5.30)	7 (6.5–9)
Glucocorticoid administration							
Oral administration	10 (12.20)	57 (34.76)	14 (43.75)	0 (0.00)	0 (00.00)	81 (28.62)	7 (6–9)
None	57 (69.51)	105 (64.02)	14 (43.75)	1 (25.00)	1 (100.00)	178 (62.90)	6 (4–8)
Unknown	15 (18.29)	2 (1.22)	4 (12.5)	3 (75.00)	0 (00.00)	24 (8.48)	3 (3–11.5)
Mutation analysis	82	160	31	4	0	277	
Deletion	56 (68.29)	106 (66.25)	20 (64.52)	2 (50.00)	0 (00.00)	184 (66.43)	6 (4–8)
Duplication	9 (10.98)	19 (11.87)	4 (12.90)	0 (0.00)	0 (00.00)	32 (11.55)	7.5 (4–9)
Point mutation	17 (20.73)	35 (21.88)	7 (22.58)	2 (50.00)	0 (00.00)	61 (22.02)	7 (4–9)

*GSGC* Gait, Stairs, Gowers’ manoeuvre, Chair, *y* year. Age with normal distributions and without normal distributions were presented as mean ± standard deviation and median (interquartile range: P25–P75), respectively

**Table 3:** Basic information of patients with Becker muscular dystrophy

		Number (%)				Total number (%)	Age (y)
	0–4 y	5–9 y	10–14 y	15–19 y	20–25 y		
Ethnic	19	44	15	7	9	94	
East Asian	19 (100.00)	44 (100.00)	15 (100.00)	7 (100.00)	9 (100.00)	94 (100.00)	7 (5–12)
Vignos scale	16 (84.21)	43 (97.73)	15 (100.00)	7 (100.00)	8 (88.89)	89 (94.68)	7 (5–12)
Brooke scale	16 (84.21)	43 (97.73)	15 (100.00)	7 (100.00)	8 (88.89)	89 (94.68)	7 (5–12)
complete GSGC scale	9 (47.37)	26 (59.09)	5 (33.33)	2 (28.57)	2 (22.22)	44 (46.81)	6.5 (5–8.5)
Gait	14 (73.68)	30 (68.18)	7 (46.67)	3 (42.86)	3 (33.33)	57 (60.64)	6 (5–9)
Time to walk 10 m	1 (5.26)	8 (18.18)	0 (0.00)	0 (100.00)	2 (22.22)	11 (11.70)	7 (5–8.5)
Climbing stairs	10 (52.63)	27 (61.36)	5 (33.33)	2 (28.57)	2 (22.22)	46 (48.94)	6.5 (5–8)
Time to climb four standard steps	3 (15.79)	8 (18.18)	0 (0.00)	0 (0.00)	2 (22.22)	13 (13.83)	5 (5–7)
Gowers’ manoeuvre	15 (78.95)	31 (70.45)	9 (60.00)	4 (57.14)	4 (44.44)	63 (67.02)	7 (5–10)
Time to stand up from ground	12 (63.16)	21 (47.73)	5 (33.33)	2 (28.57)	3 (33.33)	43 (45.74)	7 (4–9)
Arising from a chair	9 (47.37)	26 (59.09)	7 (46.67)	2 (28.57)	2 (22.22)	46 (48.94)	7 (5–9)
Time to arise from sitting	2 (10.53)	9 (20.45)	2 (13.33)	0 (0.00)	2 (22.22)	15 (15.96)	7 (5–10.5)
Loss of ambulation	0 (0.00)	0 (0.00)	0 (0.00)	0 (0.00)	0 (0.00)	0 (0.00)	–
MRI in legs	1 (5.26)	4 (9.09)	3 (20.00)	0 (0.00)	0 (0.00)	8 (8.51)	7.63 ± 4.00
Muscle biopsy	2 (10.53)	1 (2.27)	1 (6.67)	1 (14.29)	1 (11.11)	6 (6.38)	10.33 ± 7.00
Glucocorticoid administration							
Oral administration	0 (0.00)	17 (38.64)	6 (40.00)	0 (0.00)	2 (22.22)	25 (26.60)	8 (6–11)
None	11 (57.89)	26 (59.09)	6 (40.00)	1 (14.29)	1 (11.11)	45 (47.87)	7 (5–8)
Unknown	8 (42.11)	1 (2.27)	3 (20.00)	0 (0.00)	6 (66.67)	18 (19.15)	14 (3–19.5)
Mutation analysis	19	44	14	7	9	93	7 (5–12)
Deletion	12 (63.16)	32 (72.73)	7 (50.00)	4 (57.14)	6 (66.67)	61 (65.59)	7 (5–10)
Duplication	2 (10.53)	3 (6.82)	3 (21.43)	0 (0.00)	0 (0.00)	8 (8.60)	7.88 ± 3.68
Point mutation	5 (26.31)	9 (20.45)	4 (28.57)	3 (42.86)	3 (33.33)	24 (25.81)	10.10 ± 6.76

*GSGC* Gait, Stairs, Gowers’ manoeuvre, Chair, *y* year. Age with normal distributions and without normal distributions were presented as mean ± standard deviation and median (interquartile range: P25–P75), respectively

**Table 4:** Follow-up time in the longitudinal study

	Age of first examination (y)	Age of last examination (y)	Follow-up duration (y)	Frequency of examination (times)	Average interval timebetween contiguous examinations (y)	The number of individuals with 2 measurements	The number of individuals with 3 measurements	The number of individuals with 4 measurements
DMD (*n* = 84)	5.00 (3.00–7.00)	7.00 (4.00–9.00)	2.00 (1.00–3.00)	2.00 (2.00–2.00)	1.00 (1.00–2.00)	72	9	3
BMD (*n* = 29)	6.00 (3.00–9.50)	8.00 (5.50–11.50)	2.00 (1.00–3.00)	2.00 (2.00–3.00)	1.00 (1.00–2.00)	18	8	3

*DMD* Duchenne muscular dystrophy, *BMD* Becker muscular dystrophy, *y* year. Data were presented as median (interquartile range: P_25_–P_75_)

Patients whose SCRN levels were affected by renal injury (determined either by clinical manifestations or based on elevated levels of serum urea or cystatin C; serum urea of all blood samples were measured and 19 samples had results of cystatin C due to its high cost), diabetes mellitus, drug administration (angiotensin-converting-enzyme inhibitors, phlorizin, cimetidine, probenecid, or trimethoprim), obvious malnutrition (determined either by clinical manifestation or based on decreased serum albumin levels), vegetarian diet, or advanced liver disease, were excluded from the study.

### Blood examination

Blood samples were manipulated using the standard protocol in our hospital [9]. To obtain serum, five millilitres of venous blood from each participant were collected into a serum-separating tube. The time for delivering blood samples from the clinic to laboratory was within 2 h. Blood was centrifuged (at 1810 g for 5 min at room temperature) immediately after arrival in the laboratory. Then the serum top layer was transferred into an individual tube and analysed for SCRN (reference range for adults: 53–115 μmol/L; sarcosine oxidase enzymatic assay, OSR61204, Beckman Coulter, Brea, CA, USA), urea (reference range: 1.6–6.0 mmol/L for 0–3 years; 2.8–6.0 mmol/L for 4–19 years; 3.1–8.0 mmol/L for > 20 years; urease-glutamate dehydrogenase enzymatic assay, OSR6234, Beckman Coulter, Brea, CA, USA), and cystatin C levels (reference range: 0.50–1.02 mg/L; immunoturbidimetric assay, B38145, Beckman Coulter, Brea, CA, USA) in a Beckman Coulter AU5800 clinical chemistry analyser (Beckman Coulter, Brea, CA, USA). The latest follow-up blood examinations were included in the cross-sectional study to collect data in patients at the advanced stage of the disease.

For *DMD* mutation analysis, blood (2 mL) was collected and then multiplex ligation-dependent probe amplification reaction was used to detect large sequence rearrangements. Samples with negative results were checked for small mutations by next generation sequencing using an Illumina Hiseq 2000 system (Illumina Corporation, San Diego, CA, USA). The mutations we found have been reported in our previous studies [15, 16]. The following test reviewers were blinded to the results of SCRN levels.

### Motor function evaluation

Lower limb motor function was evaluated using the Vignos scale and the Gait, Stairs, Gowers’ manoeuvre, Chair (GSGC) scale [17], and upper limb motor function was evaluated using the Brooke scale just before the serum was collected [18]. Grade 7 on the Vignos scale was not used because long leg braces were not applicable in our patients and patients who could stand independently with the aid of a railing were classified as grade 8. Evaluations were limited to patients aged ≥2 years because of compliance demands. Higher grades in these scales indicated worsening motor function. The number of patients with complete GSGC scale data was smaller than that with Vignos scale data because of a demand for higher compliance for the GSGC scale. Evaluations were recorded using a Sony RX 100 Camera (Sony Corp., TYO, Japan) and the results were confirmed separately by two skilled neurologists. Data biased by poor compliance led to exclusion from the study.

### Magnetic resonance imaging

MRI results of 30 patients (DMD, 22; BMD, 8) undergoing leg MRI were included. Axial T1-weighted images (thickness: 4 mm; TR: 500 ms; TE: 22 ms) were obtained using a 1.5-T MR scanner (Achieva, Philips Cor., Amsterdam, Netherlands). Muscle fatty infiltration was evaluated by two neurologists at the maximum cross-section, with the Mercuri scale as an MRI score [19]. Leg MRI total score was the sum of MRI scores for 11 muscles in the leg, including: the vastus lateralis, rectus femoris, vastus internus, vastus medialis, adductor magnus, sartorius, adductor longus, gracilis, biceps femoris, semitendinosus, and semimembranosus muscles. Higher MRI scores indicated more severe muscle lesions.

### Muscle histology and immunohistochemistry

Muscle biopsy samples (gastrocnemius or vastus lateralis) of 21 patients were obtained (DMD, 15; BMD, 6). Hematoxylin and eosin staining and immunohistochemistry for dystrophin (anti-dystrophin antibody: NCL-DYS2 (1:50) for C terminus, NCL-DYS3 (1:50) for N terminus; Novocastra, Leica Biosystems Inc., Wetzlar, USA) were performed [20]. Muscle histology was divided into four grades using a histology scale according to fatty infiltration [21]: Grade 1. Retention of fascicular pattern with no obvious proliferation of fat or connective tissue; Grade 2. Retention of fascicular pattern plus invasion by connective tissue and/or fat proliferation; Grade 3. Disruption of muscle fascicles with marked connective tissue and/or fat proliferation; Grade 4. Severe change with replacement of more than 50% of muscle by fat and connective tissue.

Higher histological scores indicated more severe muscle lesions. The ratio of dystrophin-positive myofibers to total myofibers in the muscle section was used to evaluate dystrophin expression. All evaluations were performed separately by a pathologist and a neurologist, each with experience in muscle pathology.

### Glucocorticoid management

Glucocorticoid management consisted of oral prednisone at a dose of 0.3 mg/kg/day. Records of patients were placed in the glucocorticoid administration group if they took the glucocorticoid for at least 10 consecutive days before the SCRN test. The last dose was taken on the day immediately prior to testing because symptoms in patients with dystrophinopathy can be ameliorated after 10 days of prednisone treatment [22].

### Statistical analysis

Data were analysed using SPSS 24.0 and GraphPad Prism 6. Partial correlation was used after adjusting for age to examine correlations between SCRN levels and indexes of motor function (Vignos, Brooke, and GSGC total scores)/MRI/muscle biopsy [23]. Spearman’s correlation was used to detect correlations between SCRN levels and age.

Linear regression analysis was used to determine the differences in maturation-related changes in SCRN levels between patients with DMD, patients with BMD and controls. The dummy variables (Group1 and Group2) were used for the variable “Participant group”, because it was a nominal variable [24]. Control group was defined as (Group1 = 0, Group2 = 0); BMD group was defined as (Group1 = 0, Group2 = 1); DMD group was defined as (Group1 = 1, Group2 = 0). The variables “Age”, “Group1”, and “Group2” were included as independent variables. For achieving linearity, a log_10_ transformation was used for SCRN level, and log_10_(SCRN level) was included as the dependent variable. Given that maturation-related changes in SCRN levels potentially differed between participant groups, suggesting the interaction effect between “Age” and “Participant group”, the interaction variables “AgeGroup1” and “AgeGroup2” were included in the linear regression analysis; AgeGroup1 = Age × Group1; AgeGroup2 = Age × Group2. After the analysis (stepwise method), we chose the linear model with highest adjusted coefficient of determination (${R}_{ad}^2$), and *P* values of included variables were less than 0.05.

For the longitudinal data, the linear mixed model was used to determine changes in SCRN levels between follow-ups. According to phenotypes and changes in motor function, patients were divided into four subgroups: BMD group with undeteriorated motor function, BMD group with motor function deterioration, DMD group with undeteriorated motor function, and DMD group with motor function deterioration. The motor function deterioration was defined by increased Vignos or Brooke scores. Linear mixed models (random intercept and random slope) were constructed in R version 4.0.0 (nlme package; R Core Team, Vienna, Austria) for DMD groups and BMD group with undeteriorated motor function, taking into account the clustered nature of the data (repeated measures within patients). BMD group with motor function deterioration could not be analysed due to its low sample size (*n* = 4). These models can handle missing measurements without the need for imputation using maximum likelihood estimation. One independent variable (follow-up times, for example, this variable was defined as 1 and 2 when the patient visited our clinic for the first and second times, respectively), two covariates (age and glucocorticoid administration), and one dependent variable (SCRN level) were entered in the models. Three common covariance structures including unstructured, first-order autoregressive, and compound symmetry were examined for all groups using Akaike’s Information Criterion (AIC). The covariance structure with the lowest AIC was chosen for the analysis.

Benjamini-Hochberg procedure was used for multiple testing correction to reduce type I error in the correlation analyse. All *P* values of correlation analyses were adjusted and then false discovery rate (FDR) were shown in this study. The null hypothesis was rejected when the FDR or *P* value was less than 0.05. The data in this study were not transformed.

## Results

### SCRN levels decrease with motor function deterioration

**Fig. 1:**
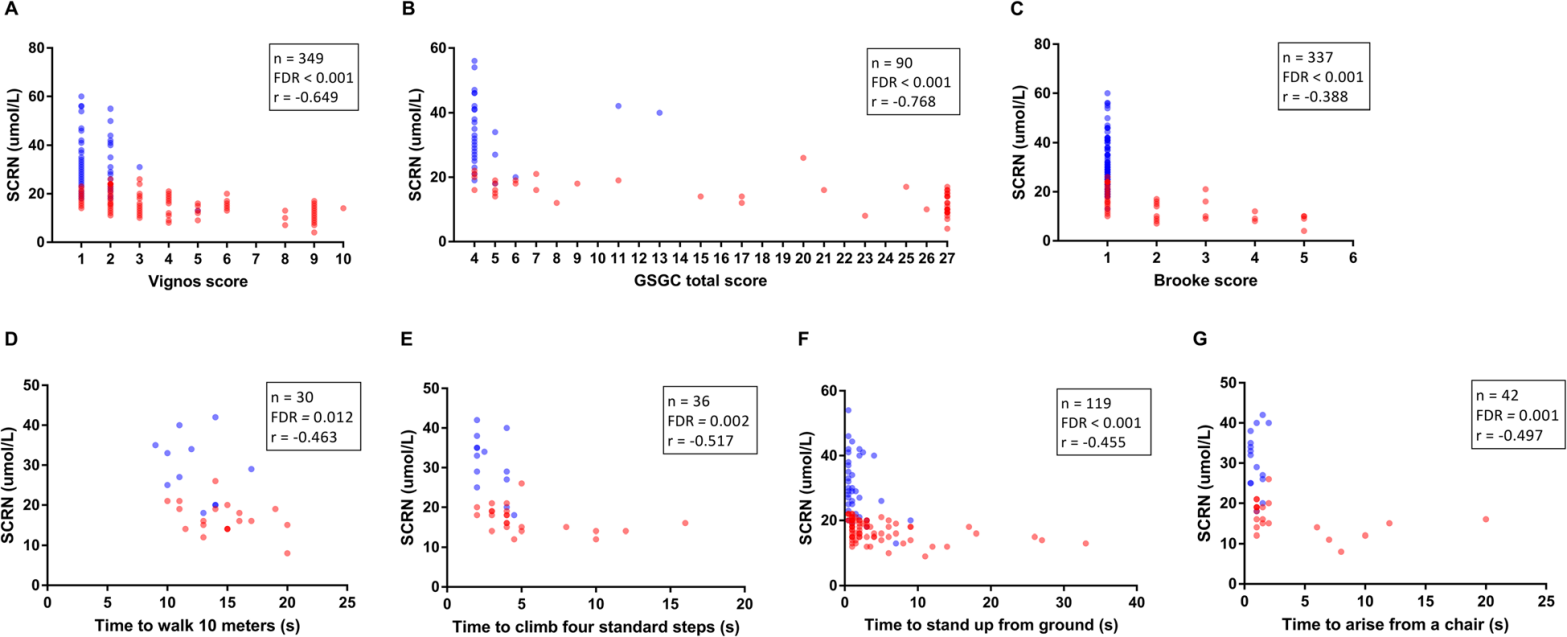
Scatter plots of serum creatinine levels versus motor function. Correlations between serum creatinine (SCRN) levels and (**a**) Vignos scores; (**b**) Gait, Stairs, Gowers’ manoeuvre, Chair (GSGC) total scores; (**c**) Brooke scores; (**d**) time to walk 10 m; (**e**) time to climb four standard steps; (**f**) time to stand up from ground; or (**g**) time to arise from a chair were adjusted for age. The color of point refers to the group of participants: red and blue denote patients with DMD and patients with BMD, respectively

All participants had normal levels of serum urea or cystatin C (supplementary material). We investigated whether the SCRN level can reflect motor function. The results indicated that SCRN levels decreased with increased Vignos scores (*n* = 349; FDR < 0.001; Fig. 1a), GSGC total scores (*n* = 90; FDR < 0.001; Fig. 1b), and Brooke scores (*n* = 337; FDR < 0.001; Fig. 1c).

The time required for movement has frequently been used as an indicator in clinical trials [17]. We found that SCRN levels decreased with increased time required to walk 10 m (*n* = 30; FDR = 0.012; Fig. 1d), climb four standard steps (*n* = 36; FDR = 0.002; Fig. 1e), stand up from the ground (*n* = 119; FDR < 0.001; Fig. 1f), and arise from a chair (*n* = 42; FDR = 0.001; Fig. 1g). Notably, patients who took a longer time to complete these movements had lower SCRN levels. Thus, SCRN levels decrease with the deterioration of limb motor function. The data in patients at different ages were shown in supplementary material.

### SCRN levels decrease with more severe muscle lesions

**Fig. 2:**
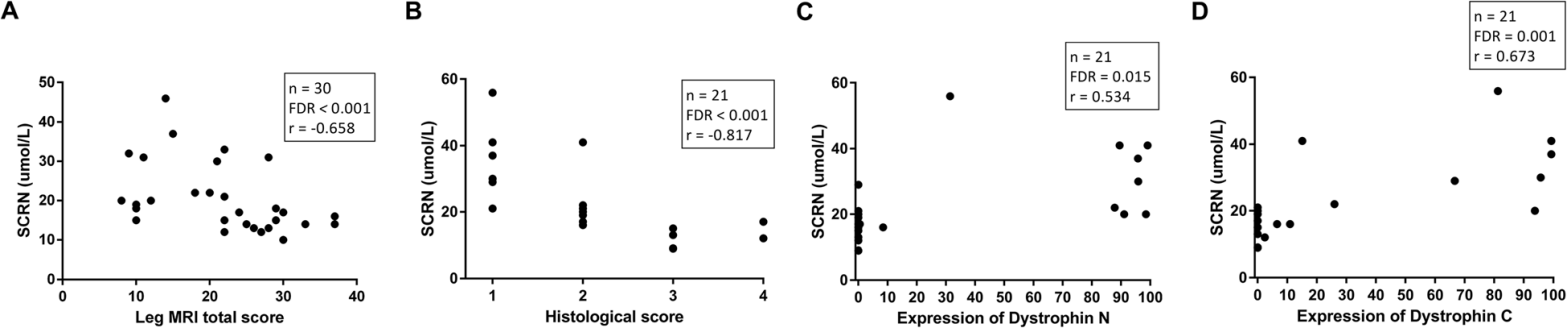
Scatter plots of serum creatinine levels versus muscle fatty infiltration and dystrophin levels. Correlations between serum creatinine (SCRN) levels and (**a**) leg MRI total scores; (**b**) histological scores; (**c**) dystrophin levels based on N-terminal; or (**d**) dystrophin levels based on C-terminal were adjusted for age. The ratio of dystrophin-positive myofibers to total myofibers (%) was used to evaluate dystrophin expression

Fatty infiltration of the muscle tissue is an important histological finding in dystrophinopathy [21]. We found that SCRN levels decreased with increased leg MRI total scores (*n* = 30; FDR < 0.001; Fig. 2a) and elevated histological scores (*n* = 21; FDR < 0.001; Fig. 2b). Thus, SCRN levels decrease with increased muscle fatty infiltration.

The dystrophin level is an important marker for clinical severity and therapeutic effectiveness [5]. We found that SCRN levels increased with higher levels of dystrophin N terminal (n = 21; FDR = 0.015; Fig. 2c) and C terminal (n = 21; FDR = 0.001; Fig. 2d).

### Different age-SCRN models between controls, patients with BMD, and patients with DMD

**Fig. 3:**
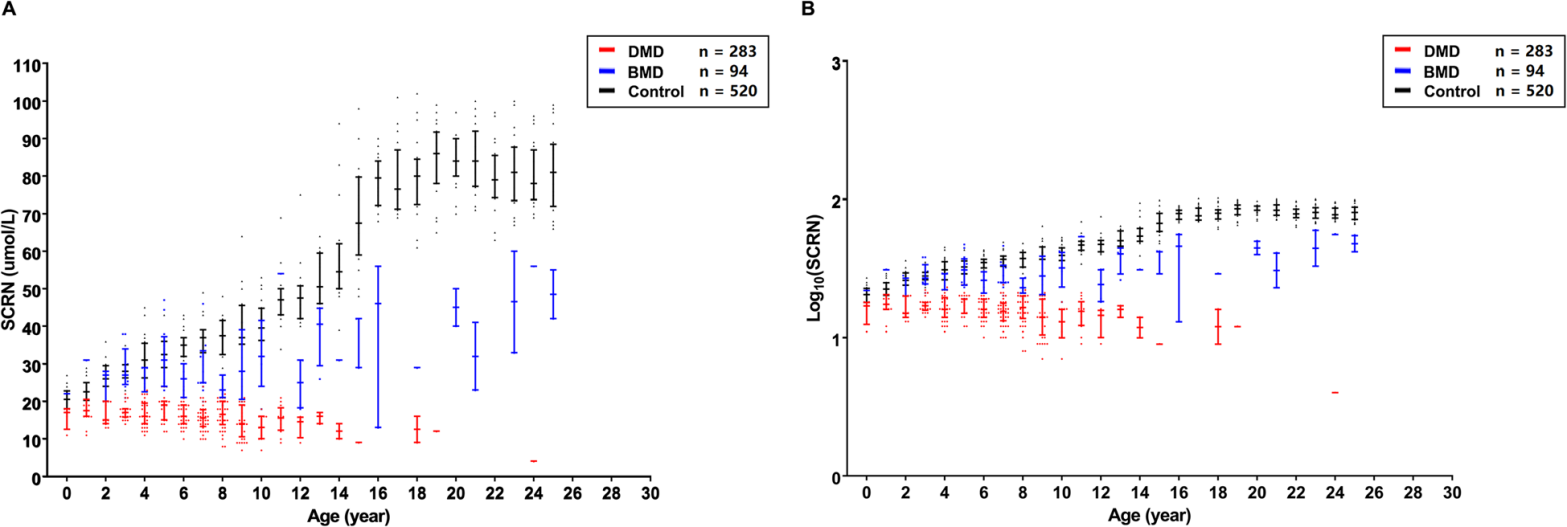
Scatter plot of serum creatinine levels versus age in participants. The association between age and (**a**) serum creatinine (SCRN) level or (**b**) Log_10_(SCRN level) in different groups. The color of point refers to the group of participants: red, blue, and black denote patients with DMD, patients with BMD, and controls, respectively. The bar shows the median and interquartile range of SCRN levels

As healthy children grow, their SCRN levels increase gradually mainly because of increased muscle mass [25]. Therefore, the maturation-related changes in SCRN levels (age-SCRN models) in dystrophinopathy need to be determined for use in clinical practice. As shown in Fig. 3a, an obvious increase in SCRN levels was observed with maturation in controls (*n* = 520; FDR < 0.001; r = 0.910). However, there were a slow increase and an overall decrease in SCRN levels with maturation in patients with BMD (*n* = 94; FDR = 0.001; r = 0.335) and DMD (*n* = 283; FDR < 0.001; r = − 0.268), respectively. Additionally, an overlap in SCRN levels between patients with BMD and control children under the age of 5 years could be observed in Fig. 3a.

Linear regression analysis was used to determine whether age-SCRN models differed significantly between groups. To achieve linearity, the dependent variable “SCRN level” was transformed to log_10_(SCRN level) (Fig. 3b). After the analysis, the model was constructed as follows:

Log_10_(SCRN level) = 1.379–0.106 × Group1 + 0.026 × Age – 0.038 × AgeGroup1–0.015 × AgeGroup2; ${R}_{ad}^2$ = 0.896.

Thus, SCRN levels were affected by age and participant group, and the age-SCRN models differed between controls, patients with BMD, and patients with DMD.

### Longitudinal analysis

**Fig. 4:**
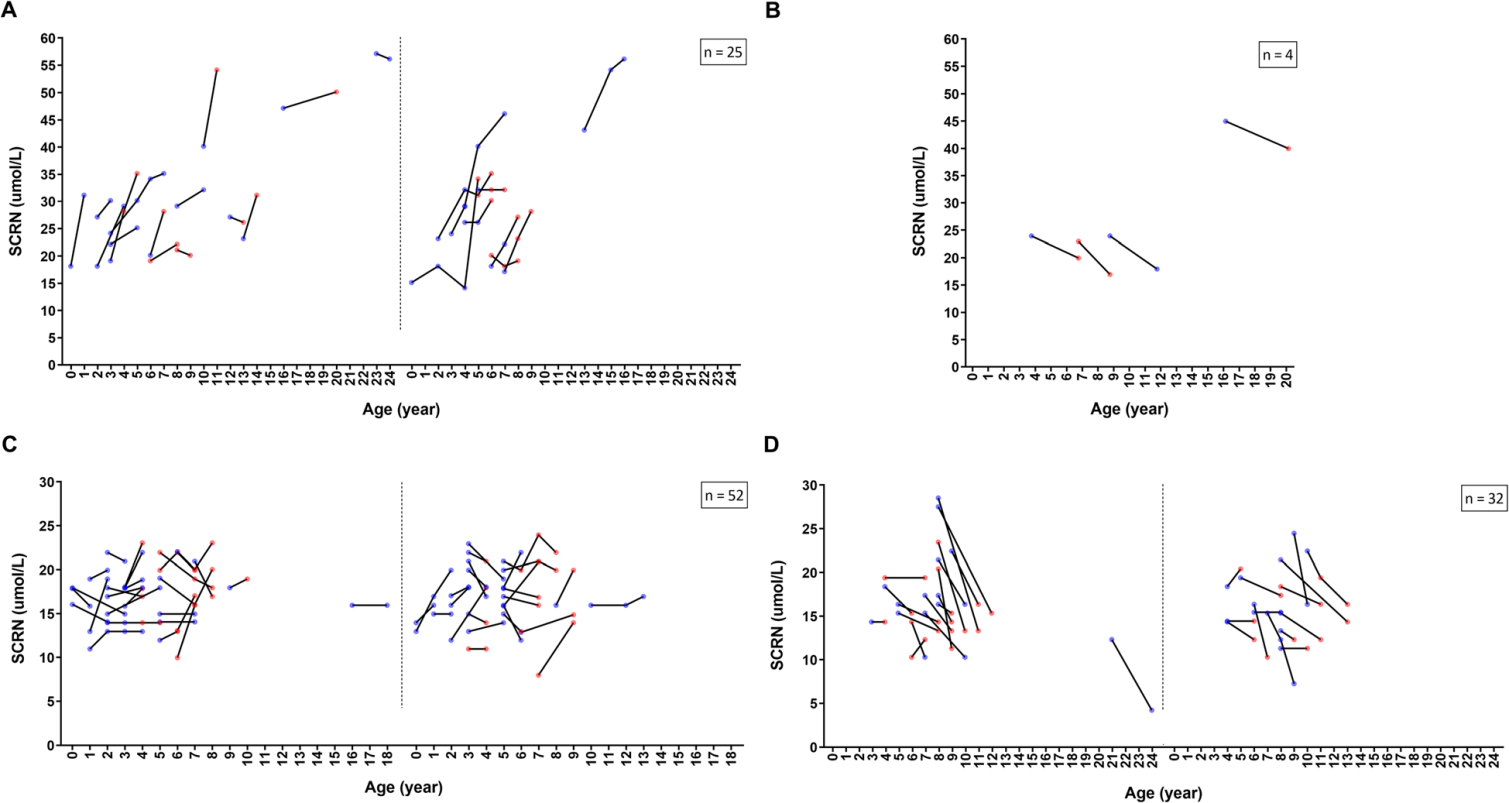
Line graphs of changes in serum creatinine levels in follow-up patients. Changes in serum creatinine (SCRN) levels in follow-up Becker muscular dystrophy (BMD) group with (**a**) undeteriorated motor function, or (**b**) deteriorated motor function and in follow-up Duchenne muscular dystrophy (DMD) group with (**c**) undeteriorated motor function, or (**d**) deteriorated motor function. A point denotes a record in a patient and the points linked by a line belong to the same patient. Increased Vignos or Brooke scores denote deteriorated motor function. The color of point refers to the glucocorticoid administration status: blue and red points denote no glucocorticoid administration and glucocorticoid administration, respectively. Graphs (**a**, **c**, and **d**) are split into two parts to decrease the overlap of points, and the graphs are thus more readable

In total, 113 patients underwent blood testing during follow-up. All participants had normal levels of serum urea (supplementary material). In BMD group with undeteriorated motor function, SCRN levels increased with maturation (*n* = 25; Coefficient = 2.210, *P* = 0.025; Fig. 4a), independent of glucocorticoid administration (*P* = 0.500). Further, SCRN levels in patients of BMD group with motor function deterioration were also shown in Fig. 4b (*n* = 4).

In DMD group with undeteriorated motor function, SCRN levels did not change with maturation (*n* = 52; *P* = 0.092; Fig. 4c), independent of glucocorticoid administration (*P* = 0.488). Additionally, decreases in SCRN levels were observed in DMD group with motor function deterioration (*n* = 32; Coefficient = − 3.416, *P* = 0.001; Fig. 4d), independent of glucocorticoid administration (*P* = 0.422). Thus, changes in SCRN levels reflect changes in motor function in dystrophinopathy, independent of glucocorticoid administration.

## Discussion

In this study, we evaluated whether the SCRN level can reflect the progression of dystrophinopathy. We found that decreased SCRN levels correlated with motor function deterioration in dystrophinopathy, and the findings validate those in our previous preliminary study [10]. The longitudinal data presented here confirm that changes in SCRN levels are associated with motor function changes. The correlation between SCRN levels and severity of muscle lesions was also demonstrated. Furthermore, the maturation-related changes in SCRN levels differed between patients and controls. A marked increase in SCRN levels with maturation in controls conforms to previous findings [25]; the difference in age-SCRN models between DMD and controls conforms to that result in a previous study assessing blood metabolomics in patients with DMD [26]. Thus, the SCRN level can be used to assess disease progression in dystrophinopathy.

SCRN levels in patients with DMD rarely overlap with those in control individuals; however, there was an overlap in SCRN levels between patients with BMD and control children under the age of 5 years, which is probably related to the age of onset of motor dysfunction in patients with BMD (above 5 years). SCRN levels increased more slowly with maturation in patients with BMD aged > 5 years than in controls, indicating increased muscle injury in patients with BMD. Therefore, SCRN levels are highly correlated with disease progression. Moreover, the present results validate our previous findings that SCRN levels can distinguish DMD from BMD [9].

The creatine and phosphocreatine system is essential as an energy-supporting mechanism in muscle tissues, where creatine is primarily found (up to 94% of total creatine in the body) [27]. Creatine and phosphocreatine in myofibers are converted non-enzymatically to creatinine at a stable rate, which is then released into the blood as SCRN [28]. Thus, SCRN levels are correlated with body muscle mass (BMM) and have thus been used to estimate BMM [29, 30]. Decreased SCRN levels in dystrophinopathy probably result from decreased BMM, and the hypothesis is supported by our findings that SCRN levels correlate with the severity of muscle lesions. However, it remains unclear whether the creatinine level can be used as a precise method for measuring BMM in dystrophinopathy [31–34]. Furthermore, a recent study showed that SCRN could be a biomarker in spinal muscular atrophy, a disease also characterized by muscle wasting and weakness, as well as multiple forms of muscular dystrophy [35–37]. Thus, this suggests that SCRN may be a potential biomarker in neuromuscular disorders, and its applicability in other diseases should be further investigated.

MRI findings/dystrophin levels have been used to assess therapeutic effects [5, 38]. The present study demonstrated correlations between SCRN levels and MRI findings/dystrophin levels, suggesting that assessing SCRN levels might be a suitable alternative for evaluating therapeutic effects. Furthermore, the differences in age-SCRN model between controls, patients with BMD, and patients with DMD indicate that the age-SCRN model could be recovered (i.e., from the DMD to BMD model; from the DMD to normal model; from the BMD to normal model) if the treatment leads to the recovery of injured muscles. Therefore, a continuous, long-term evaluation of the SCRN level could facilitate the assessment of disease progression and therapeutic effects. Furthermore, although glucocorticoids, a common medication used in patients with dystrophinopathy, have a mild effect (10% increase) on SCRN level [1, 39], this effect would not limit its application for monitoring disease progress in dystrophinopathy because changes in SCRN levels were highly associated with motor function changes, independent of glucocorticoid administration.

Combined with our previous study [9], SCRN has two application scenarios in dystrophinopathy by reflecting the muscle injury severity: distinguishing phenotypes and assessing disease progression. Thus, SCRN is a potential prognostic biomarker as well as a surrogate outcome measure. Additionally, these two applications can also be combined. For example, exon skipping therapy is suitable for DMD phenotype; after confirming DMD phenotype with the help of SCRN level, the changes in SCRN levels in the follow-ups could reflect the response to the treatment.

This study is not without limitations. The assessment accuracy of the SCRN level changes in patients under the age of 1 year was not clear, because few such patients were diagnosed. Additionally, SCRN levels in patients with advanced BMD were unknown; therefore, a longer-term follow-up study is needed to investigate this issue. Our observations in patients with DMD suggest that SCRN levels in patients with advanced BMD probably decrease as motor function deteriorates. And the linear regression model in the age range > 14 years might not be accurate because of the low sample size (DMD: 5; BMD: 16), which needs the further verification. Moreover, kidney damage was reported in patients with Duchenne muscular dystrophy and renal dysfunction may affect SCRN levels [32]; thus, it is necessary to evaluate renal function when using SCRN levels to assess dystrophinopathy progression and therapeutic responses.

## Conclusions

SCRN levels could be used to assess disease progression in patients with dystrophinopathy. The approach assessing the SCRN level is inexpensive, simple, and minimally invasive and has been extensively used in clinical settings; therefore, SCRN is a promising biomarker for assessing disease progression of dystrophinopathy and could be a potential surrogate outcome measure in clinical trials.

## Supplementary Information

**Additional file 1 **: **Fig. S1.** Renal function indexes in participants in the cross-sectional study. **Fig. S2**. Scatter plots of serum creatinine levels versus motor function scores in patients at different ages. **Fig. S3**. Scatter plots of serum creatinine levels versus time required for movement in patients at different ages. **Fig. S4**. Serum urea levels in patients in the longitudinal study.

## Data Availability

The datasets used and/or analysed during the current study are available from the corresponding author on reasonable request.

## Abbreviations

DMD: Duchenne muscular dystrophy
BMD: Becker muscular dystrophy
SCRN: Serum creatinine
MRI: Magnetic resonance imaging
IMD: Intermediate muscular dystrophy
GSGC: Gait, Stairs, Gowers’ manoeuvre, Chair
${R}_{ad}^2$: Adjusted coefficient of determination
AIC: Akaike’s Information Criterion
FDR: False discovery rate
BMM: Body muscle mass

## References

[1] Flanigan KM (2014). Duchenne and Becker muscular dystrophies. Neurol Clin.

[2] Fairclough RJ, Bareja A, Davies KE (2011). Progress in therapy for Duchenne muscular dystrophy. Exp Physiol.

[3] Angelini C, Pegoraro E, Turella E, Intino MT, Pini A, Costa C (1994). Deflazacort in Duchenne dystrophy: study of long-term effect. Muscle Nerve.

[4] Hathout Y, Seol H, Han MH, Zhang A, Brown KJ, Hoffman EP (2016). Clinical utility of serum biomarkers in Duchenne muscular dystrophy. Clin Proteomics.

[5] Wilton SD, Fletcher S, Flanigan KM (2014). Dystrophin as a therapeutic biomarker: are we ignoring data from the past?. Neuromuscul Disord.

[6] Fischer D, Hafner P, Rubino D, Schmid M, Neuhaus C, Jung H, Bieri O, Haas T, Gloor M, Fischmann A, Bonati U (2016). The 6-minute walk test, motor function measure and quantitative thigh muscle MRI in Becker muscular dystrophy: a cross-sectional study. Neuromuscul Disord.

[7] Aartsma-Rus A, Ferlini A, Vroom E (2014). Biomarkers and surrogate endpoints in Duchenne: meeting report. Neuromuscul Disord.

[8] Zocevic A, Rouillon J, Wong B, Servais L, Voit T, Svinartchouk F (2015). Evaluation of the serum matrix metalloproteinase-9 as a biomarker for monitoring disease progression in Duchenne muscular dystrophy. Neuromuscul Disord.

[9] Wang L, Chen M, He R, Sun Y, Yang J, Xiao L, Cao J, Zhang H, Zhang C (2017). Serum creatinine distinguishes Duchenne muscular dystrophy from Becker muscular dystrophy in patients aged <=3 years: a retrospective study. Front Neurol.

[10] Zhang H, Zhu Y, Sun Y, Liang Y, Li Y, Zhang Y, Deng L, Wen X, Zhang C (2015). Serum creatinine level: a supplemental index to distinguish Duchenne muscular dystrophy from Becker muscular dystrophy. Dis Markers.

[11] Wang L, Xu M, Li H, Zhang C (2018). Reasons for first visit to neurologists in Chinese patients with dystrophinopathy: a survey study. Neuromuscul Disord.

[12] Mah JK, Korngut L, Dykeman J, Day L, Pringsheim T, Jette N (2014). A systematic review and meta-analysis on the epidemiology of Duchenne and Becker muscular dystrophy. Neuromuscul Disord.

[13] Marden FA, Connolly AM, Siegel MJ, Rubin DA (2005). Compositional analysis of muscle in boys with Duchenne muscular dystrophy using MR imaging. Skelet Radiol.

[14] Romitti PA, Zhu Y, Puzhankara S, James KA, Nabukera SK, Zamba GK (2015). Prevalence of Duchenne and Becker muscular dystrophies in the United States. Pediatrics.

[15] Wang L, Xu M, Li H, He R, Lin J, Zhang C, Zhu Y (2019). Genotypes and phenotypes of DMD small mutations in Chinese patients with Dystrophinopathies. Front Genet.

[16] Yang J, Li SY, Li YQ, Cao JQ, Feng SW, Wang YY, Zhan YX, Yu CS, Chen F, Li J, Sun XF, Zhang C (2013). MLPA-based genotype-phenotype analysis in 1053 Chinese patients with DMD/BMD. BMC Med Genet.

[17] Angelini C (2007). The role of corticosteroids in muscular dystrophy: a critical appraisal. Muscle Nerve.

[18] Jung IY, Chae JH, Park SK, Kim JH, Kim JY, Kim SJ, Bang MS (2012). The correlation analysis of functional factors and age with Duchenne muscular dystrophy. Ann Rehabil Med.

[19] Bing Q, Hu K, Tian Q, Zhao Z, Shen H, Li N (2016). Semi-quantitative assessment of lower limb MRI in dystrophinopathy. Int J Clin Exp Med.

[20] Na SJ, Kim WJ, Kim SM, Lee KO, Yoon B, Choi YC (2013). Clinical, immunohistochemical, Western blot, and genetic analysis in dystrophinopathy. J Clin Neurosci.

[21] Kinali M, Arechavala-Gomeza V, Cirak S, Glover A, Guglieri M, Feng L, Hollingsworth KG, Hunt D, Jungbluth H, Roper HP, Quinlivan RM, Gosalakkal JA, Jayawant S, Nadeau A, Hughes-Carre L, Manzur AY, Mercuri E, Morgan JE, Straub V, Bushby K, Sewry C, Rutherford M, Muntoni F (2011). Muscle histology vs MRI in Duchenne muscular dystrophy. Neurology..

[22] Griggs RC, Moxley RT, Mendell JR, Fenichel GM, Brooke MH, Pestronk A (1991). Prednisone in Duchenne dystrophy. A randomized, controlled trial defining the time course and dose response. Clinical investigation of Duchenne dystrophy group. Arch Neurol.

[23] Ionas E (2020). Partial correlations in compositional data analysis. Applied Computing and Geosciences.

[24] Suits DB (1957). Use of dummy variables in regression equations. J Am Stat Assoc.

[25] Uemura O, Honda M, Matsuyama T, Ishikura K, Hataya H, Yata N, Nagai T, Ikezumi Y, Fujita N, Ito S, Iijima K, Kitagawa T (2011). Age, gender, and body length effects on reference serum creatinine levels determined by an enzymatic method in Japanese children: a multicenter study. Clin Exp Nephrol.

[26] Boca SM, Nishida M, Harris M, Rao S, Cheema AK, Gill K, Seol H, Morgenroth LP, Henricson E, McDonald C, Mah JK, Clemens PR, Hoffman EP, Hathout Y, Madhavan S (2016). Discovery of metabolic biomarkers for Duchenne muscular dystrophy within a natural history study. PLoS One.

[27] Wyss M, Kaddurah-Daouk R (2000). Creatine and creatinine metabolism. Physiol Rev.

[28] Heymsfield SB, Arteaga C, McManus C, Smith J, Moffitt S (1983). Measurement of muscle mass in humans: validity of the 24-hour urinary creatinine method. Am J Clin Nutr.

[29] Thongprayoon C, Cheungpasitporn W, Kashani K (2016). Serum creatinine level, a surrogate of muscle mass, predicts mortality in critically ill patients. J Thorac Dis.

[30] Kim SW, Jung HW, Kim CH, Kim KI, Chin HJ, Lee H (2016). A new equation to estimate muscle mass from creatinine and cystatin C. PLoS One.

[31] Franciotta D, Zanardi MC, Albertotti L, Orcesi S, Berardinelli A, Pichiecchio A, Uggetti C, Tagliabue A (2003). Measurement of skeletal muscle mass in Duchenne muscular dystrophy: use of 24-h creatinine excretion. Acta Diabetol.

[32] Braat E, Hoste L, De Waele L, Gheysens O, Vermeersch P, Goffin K (2015). Renal function in children and adolescents with Duchenne muscular dystrophy. Neuromuscul Disord.

[33] Viollet L, Gailey S, Thornton DJ, Friedman NR, Flanigan KM, Mahan JD, Mendell JR (2009). Utility of cystatin C to monitor renal function in Duchenne muscular dystrophy. Muscle Nerve.

[34] Hankard R, Gottrand F, Turck D, Carpentier A, Romon M, Farriaux JP (1996). Resting energy expenditure and energy substrate utilization in children with Duchenne muscular dystrophy. Pediatr.

[35] Alves CRR, Zhang R, Johnstone AJ, Garner R, Nwe PH, Siranosian JJ, Swoboda KJ (2020). Serum creatinine is a biomarker of progressive denervation in spinal muscular atrophy. Neurology..

[36] Spitali P, Hettne K, Tsonaka R, Sabir E, Seyer A, Hemerik JBA, Goeman JJ, Picillo E, Ergoli M, Politano L, Aartsma-Rus A (2018). Cross-sectional serum metabolomic study of multiple forms of muscular dystrophy. J Cell Mol Med.

[37] Harris E, Bladen CL, Mayhew A, James M, Bettinson K, Moore U (2016). The Clinical Outcome Study for dysferlinopathy: An international multicenter study. Neurol Genet.

[38] Arpan I, Willcocks RJ, Forbes SC, Finkel RS, Lott DJ, Rooney WD, Triplett WT, Senesac CR, Daniels MJ, Byrne BJ, Finanger EL, Russman BS, Wang DJ, Tennekoon GI, Walter GA, Sweeney HL, Vandenborne K (2014). Examination of effects of corticosteroids on skeletal muscles of boys with DMD using MRI and MRS. Neurology.

[39] Andreev E, Koopman M, Arisz L (1999). A rise in plasma creatinine that is not a sign of renal failure: which drugs can be responsible?. J Intern Med.

