# Supplementary material for: Serum creatinine as a biomarker for dystrophinopathy: a cross-sectional and longitudinal study
Source: BMC Neurol. 2021 Sep 25;21:372. doi: 10.1186/s12883-021-02382-7 (PMC8464115; doi:10.1186/s12883-021-02382-7)
Supplement: Supplementary file 1 — Additional file 1 : Fig. S1. Renal function indexes in participants in the cross-sectional study. Fig. S2. Scatter plots of serum creatinine levels versus motor function scores in patients at different ages. Fig. S3. Scatter plots of serum creatinine levels versus time required for movement in patients at different ages. Fig. S4. Serum urea levels in patients in the longitudinal study. [file 12883_2021_2382_MOESM1_ESM.docx]

**Serum creatinine as a biomarker for dystrophinopathy: a cross-sectional and longitudinal study**

-Supplementary material-

Liang Wang^1^, Min Xu^2^, Dawei Liu^3^, Yingyin Liang^1^, Pinning Feng^4^, Huan Li^1^, Yuling Zhu^1^, Ruojie He^1^, Jinfu Lin^1^, Huili Zhang^5^, Ziyu Liao^1^, Cheng Zhang^1, *^

^1^ Department of Neurology, The First Affiliated Hospital, Sun Yat-sen University, Guangdong Provincial Key Laboratory of Diagnosis and Treatment of Major Neurological Diseases, National Key Clinical Department and Key Discipline of Neurology. No. 58 Zhongshan Road 2, Guangzhou, 510080, China.

^2^ Department of Dermatology, The Second Affiliated Hospital of Guangzhou Medical University. No. 250 Changgang East Road, Guangzhou, 510260, China

^3^ Department of Pathology, The First Affiliated Hospital, Sun Yat-sen University. No. 58 Zhongshan Road 2, Guangzhou, 510080, China.

^4^ Department of Laboratory, The First Affiliated Hospital, Sun Yat-sen University. No. 58 Zhongshan Road 2, Guangzhou, 510080, China.

^5^ Department of Neurology, Guangzhou First People’s Hospital. No. 1 Panfu Road, Guangzhou, 510180, China


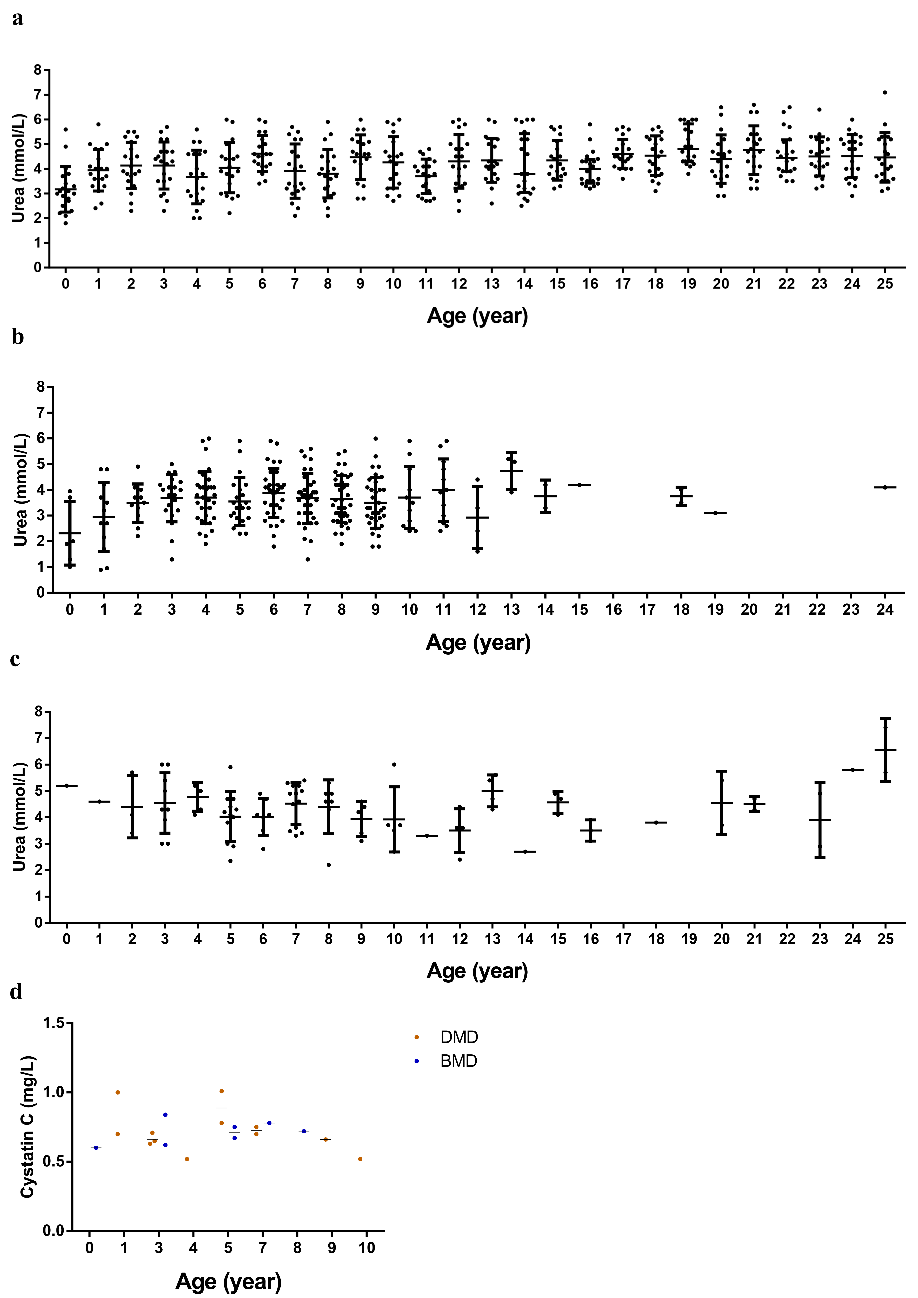


**Figure S1. Renal function indexes in participants in the cross-sectional study.** (a-c) Serum urea levels in (a) controls, (b) patients with Duchenne muscular dystrophy, and (c) patients with Becker muscular dystrophy. (d) Serum cystatin C levels in 19 patients with dystrophinopathy.


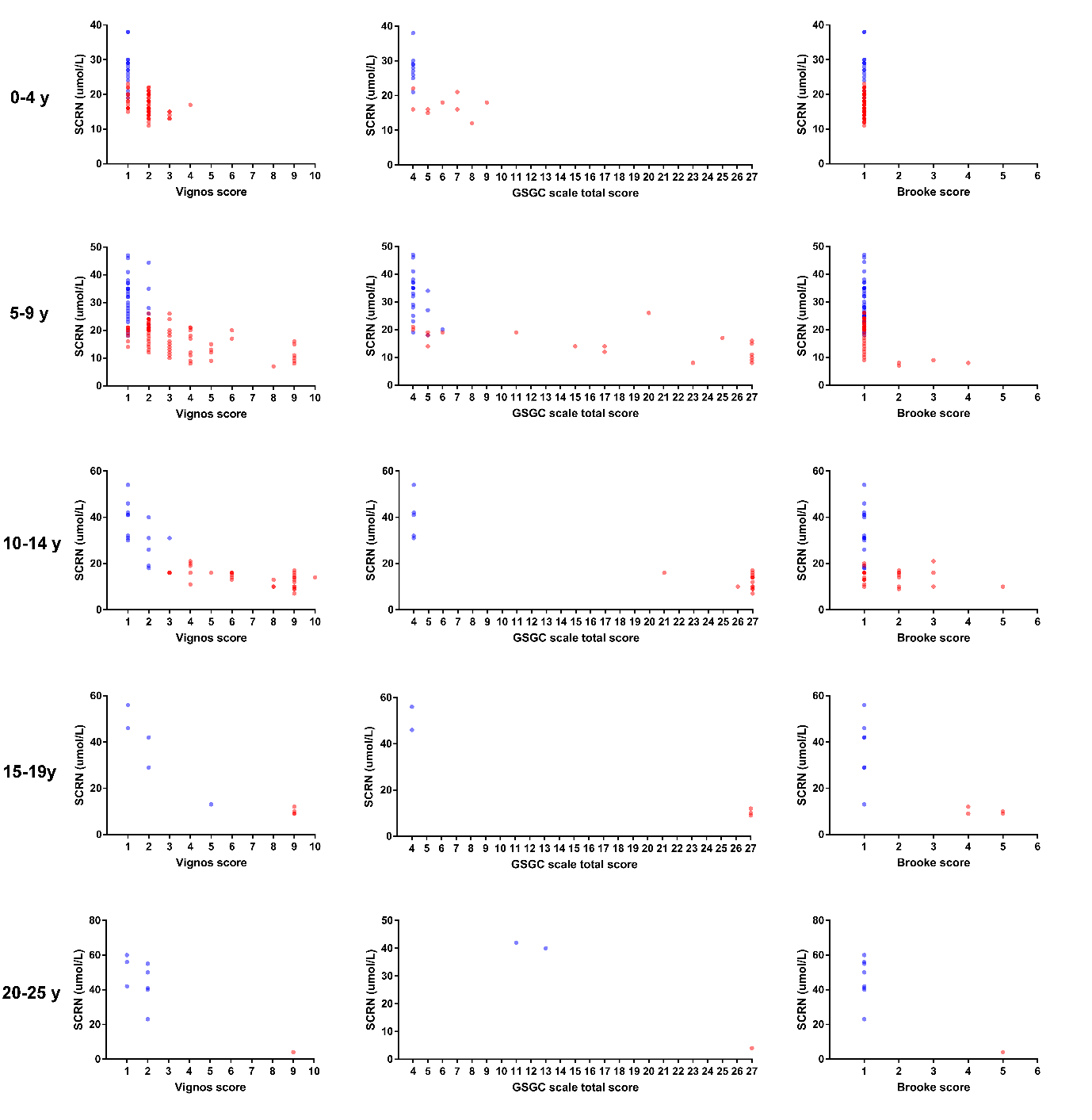


**Figure S2. Scatter plots of serum creatinine levels versus motor function scores in patients at different ages.** The red and blue points denote DMD and BMD, respectively.


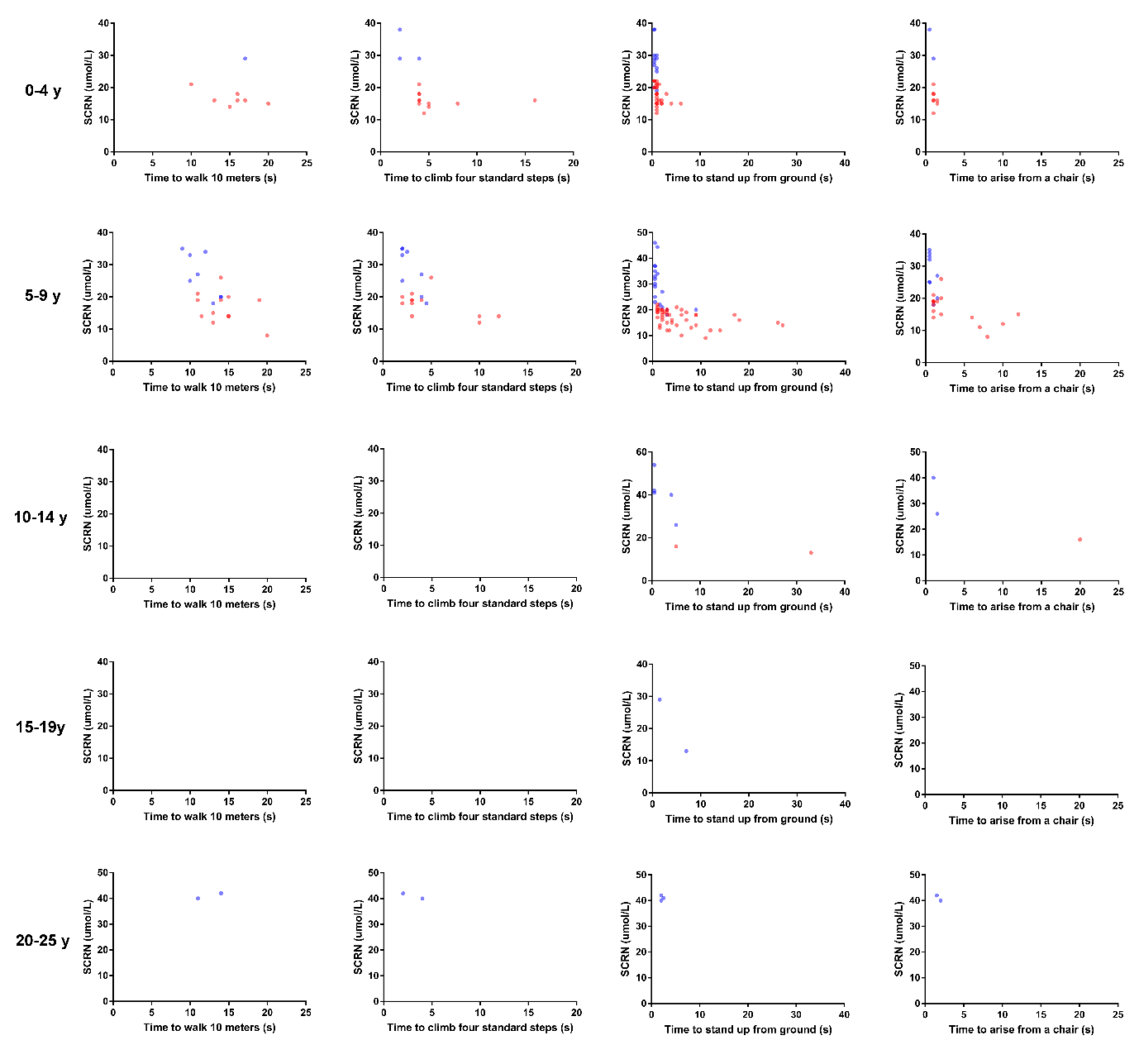


**Figure S3. Scatter plots of serum creatinine levels versus time required for movement in patients at different ages.** The red and blue points denote DMD and BMD, respectively.


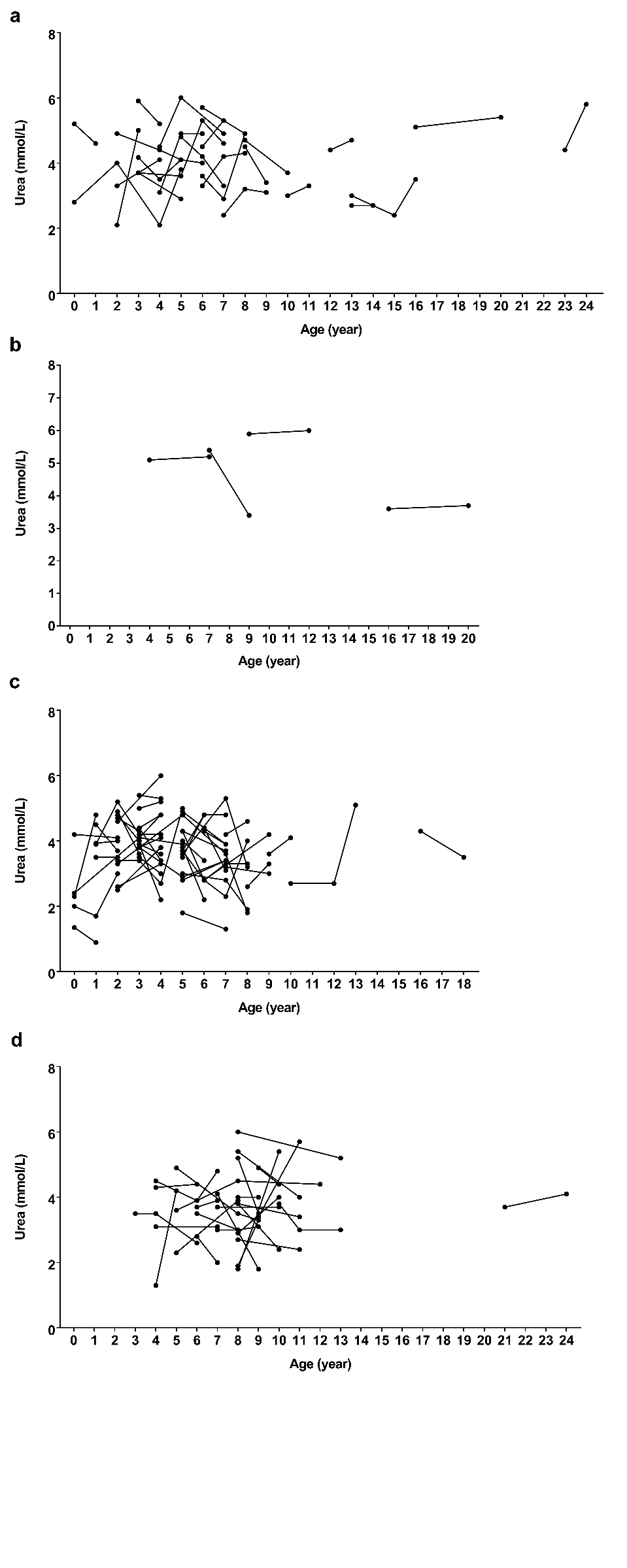


**Figure S4. Serum urea levels in patients in the longitudinal study.** Changes in serum urea in follow-up Becker muscular dystrophy group with (a) undeteriorated motor function, or (b) deteriorated motor function and in follow-up Duchenne muscular dystrophy group with (c) undeteriorated motor function, or (d) deteriorated motor function.
